# Prevalence rate, predictors and long-term course of probable posttraumatic stress disorder after major trauma: a prospective cohort study

**DOI:** 10.1186/1471-244X-12-236

**Published:** 2012-12-27

**Authors:** Juanita A Haagsma, Akkie N Ringburg, Esther MM van Lieshout, Ed F van Beeck, Peter Patka, Inger B Schipper, Suzanne Polinder

**Affiliations:** 1 Department of Public Health, Erasmus MC, University Medical Center Rotterdam, P.O. Box 2040, Rotterdam, 3000, CA, The Netherlands; 2 Department of Surgery–Traumatology, Erasmus MC, University Medical Center Rotterdam, P.O. Box 2040, Rotterdam, 3000, CA, the Netherlands; 3 Department of Surgery-Traumatology, Leiden University Medical Center, P.O. Box 9600, Leiden, 2300, RC, the Netherlands; 4 Department of Surgery, Ikazia Hospital, Montessoriweg 1, Rotterdam, 3083, AN, The Netherlands

**Keywords:** Major trauma, Posttraumatic stress disorder, Follow-up study

## Abstract

**Background:**

Among trauma patients relatively high prevalence rates of posttraumatic stress disorder (PTSD) have been found. To identify opportunities for prevention and early treatment, predictors and course of PTSD need to be investigated. Long-term follow-up studies of injury patients may help gain more insight into the course of PTSD and subgroups at risk for PTSD. The aim of our long-term prospective cohort study was to assess the prevalence rate and predictors, including pre-hospital trauma care (assistance of physician staffed Emergency Medical Services (EMS) at the scene of the accident), of probable PTSD in a sample of major trauma patients at one and two years after injury. The second aim was to assess the long-term course of probable PTSD following injury.

**Methods:**

A prospective cohort study was conducted of 332 major trauma patients with an Injury Severity Score (ISS) of 16 or higher. We used data from the hospital trauma registry and self-assessment surveys that included the Impact of Event Scale (IES) to measure probable PTSD symptoms. An IES-score of 35 or higher was used as indication for the presence of probable PTSD.

**Results:**

One year after injury measurements of 226 major trauma patients were obtained (response rate 68%). Of these patients 23% had an IES-score of 35 or higher, indicating probable PTSD. At two years after trauma the prevalence rate of probable PTSD was 20%. Female gender and co-morbid disease were strong predictors of probable PTSD one year following injury, whereas minor to moderate head injury and injury of the extremities (AIS less than 3) were strong predictors of this disorder at two year follow-up. Of the patients with probable PTSD at one year follow-up 79% had persistent PTSD symptoms a year later.

**Conclusions:**

Up to two years after injury probable PTSD is highly prevalent in a population of patients with major trauma. The majority of patients suffered from prolonged effects of PTSD, underlining the importance of prevention, early detection, and treatment of injury-related PTSD.

## Background

Major trauma, which can be defined as an injury with an Injury Severity Score (ISS) of 16 or higher, has a large impact, not in the least because of the relatively young age of the average severely injured patient [1]. A large proportion of the severely injured patients report significantly reduced health-related quality of life with functional limitations years after trauma [2-4]. In rehabilitation, the main focus lies in the treatment of physical injuries. Nonetheless, over the past decades the importance of psychological morbidity continued to gain attention, specifically concerning posttraumatic stress disorder (PTSD).

PTSD may result from any event that involves an injury, or threatened or actual death (of others). PTSD symptoms are characterized by re-experiencing, avoidance and hyper arousal, and may either appear immediately after the event or have a delayed onset [5]. In the general population PTSD prevalence rates between 2-4% have been found [6,7]. Trauma patients have relatively high prevalence rates of PTSD; prevalence rates up to 39% have been found one to four months after the injury [8]. At long-term follow-up (>1 year) PTSD prevalence rates vary from 5% among traffic injury victims [9] to 32% among major trauma patients [10].

Predictors of PTSD following major trauma are gender, age, presence of chronic illnesses, cause of injury, coping style, pain, cognitive functioning when discharged from the hospital and employment [11-13]. To our knowledge, the effect of pre-hospital trauma care (i.e. assistance of physician staffed Emergency Medical Services (EMS) at the scene of the accident) on the risk of developing PTSD has not yet been studied. Identifying subgroups at risk for PTSD is important for the targeting of PTSD prevention and to facilitate early treatment when PTSD has developed. Research has shown that PTSD can be effectively treated at an early stage [14]. However, symptoms of PTSD may not always develop immediately after the injury. In some cases, symptoms develop relatively long after sustaining the trauma. This time delay between the injury and PTSD may hamper identification of risk groups. Follow-up studies of injury patients may help gain more insight into the long-term course of PTSD and subgroups at risk for PTSD.

### Aim of this study

The primary aim of our study was to assess the prevalence rate and predictors of probable PTSD in a sample of major trauma patients at one and two years after injury. In addition to the influence of socio-demographic, physical and injury related factors, we explored the association of pre-hospital trauma care, i.e., the presence versus absence of pre-hospital trauma care at the scene of the accident via assistance of physician staffed helicopter or other EMS teams. Secondly, this study aimed to assess the long-term course of probable PTSD following injury.

## Methods

### Study population and design

From January 2004 until July 2006, a prospective cohort study was conducted, including all consecutive major trauma patients with an Injury Severity Score (ISS) [15] of 16 or higher and aged 16 years or older, that were presented to a level I trauma center in a Dutch trauma region serving 4.9 million inhabitants. Patients that were pronounced Dead On Arrival were excluded. For the purpose of this study part of the data were derived from the Hospital Trauma Registry that documents the same variables as the Major Trauma Outcome Study database [16] (i.e., Age, Glasgow Coma Scale [17], Revised Trauma Score [18], Mechanism Of Injury, and injury specifics such as the Injury Severity Score (ISS)). Missing data were obtained from the original ambulance charts. This PTSD study was part of a prospective cost effectiveness analysis of (helicopter) emergency medical services in the Netherlands [19]. One and two years after trauma all patients received a questionnaire. In absence of response patients received a phone call one month after the mailing in order to increase participation.

This study was conducted with the approval of the Ethics Committee Erasmus MC University Hospital and carried out in compliance with the Helsinki Declaration. All patients, or in case of pediatric patients, their parents of guardian, provided informed consent.

### Impact of event scale

The impact of event scale (IES) may be used to assess symptoms of posttraumatic stress indicative of PTSD [20]. The IES is a self-report questionnaire that consists of 15 items, which measure intrusive re-experiences of the trauma and avoidance of trauma-related stimuli. The IES measures only two of the three main PTSD symptoms, namely intrusion and avoidance. By combining the 15 items the total IES-score, ranging from 0 through 75, can be calculated. Wohlfarth et al. showed that a cut-off score of 35 on the total IES-score produced sensitivity of .89, specificity of .94 when the DSM-IV was used as the diagnostic criteria for PTSD [21]. Therefore, we assumed an IES-score higher than 35 (IES ≥ 34) signifies symptoms of posttraumatic stress indicative of PTSD. Because the IES measures only two of the three main PTSD symptoms, the IES cannot be used to assess PTSD. Therefore, we indicate cases with an IES-score higher than 34 as cases with probable PTSD. The Dutch translation of the IES has been found to be valid and reliable [22].

### Socio-demographic, injury, and health care related characteristics

From the literature, potential determinants of functional outcome were identified [23-25]. These determinants of functional outcome were grouped into socio-demographic (age and gender, education level, household composition, and pre-existing co-morbid illnesses), injury (ISS, Revised Trauma Score (RTS), and injury location), and health care related characteristics (HEMS (Helicopter Emergency Medical Services) or EMS). For the purpose of this study, several socio-demographic variables were grouped into two categories. Education was subdivided into primary school level or higher. Household composition was subdivided into households existing of a single person or more persons.

Co-morbidity is defined as the presence of any co-existing medical diseases or disease processes additional to the injury that the injury patients sustained [26]. The following diseases were assessed as co-morbid disease: asthma, chronic bronchitis, chronic non-specific lung disease; heart disease; diabetes; back hernia or chronic backache; osteoarthritis; rheumatoid arthritis and cancer.

Co-morbidity was subdivided into three groups. A co-morbid condition was defined as a disease that existed at the time of trauma according to the patient or the family. Co-morbidity was categorised into a group without pre-existing disease, a group with one co-morbid disease and thirdly, a group with two or more co-morbidities.

The injury diagnosis was verified at the individual level with information from the hospital discharge register according to the *Abbreviated Injury Scale*, *1990 Revision*, *update 1998*[27].

### Pre-hospital trauma care

Pre-hospital trauma care was upgraded in the Netherlands in 1995, when physician staffed HEMS were introduced in addition to nurse staffed EMS. For all major trauma patients in this study it was registered which type of pre-hospital care (HEMS or EMS) was provided.

### Statistical analysis

The statistical analyses were performed using the Statistical Package for the Social Sciences (SPSS) version 12.0 (SPSS, Chicago, IL, USA). We calculated the IES-score of each of the injury patients. The IES-score can only be calculated if all IES items are completed. In 4.5% of the cases data of one or two of the 15 IES items were missing. For these cases, the missing IES item was estimated by calculating the median value of 5 nearby points. The missing data was then imputed by the estimated values [28]. Fisher’s exact tests were used to test for differences between the study population and respondents. Non-parametric variables (age, Glasgow coma Score, RTS, and ISS) were tested using the Mann–Whitney U-test.

Chi-square statistics (dichotomous variables) and Student t tests (continuous variables) were used to test for differences between injury patients with IES scores higher or lower than 35.

Univariate logistic regression analyses were used to determine the predictive value of socio-demographics, presence of co-morbid diseases, sustained injuries and pre-hospital trauma with regard to probable PTSD (IES ≥ 35).

To dichotomize severity level, the ISS were categorized into two classes (16–24 versus ≥25). Also, we dichotomized the injuries in each body region (<3 versus ≥3).

Stepwise multiple regression analyses (enter method) was applied to investigate the association between demographics (block 1), hospitalization and comorbidity (block 2) posttraumatic stress symptoms indicative of PTSD (IES ≥ 35) (block 3). Variables with a p-value ≤ 0.1 in the univariate analysis were applied in the multivariate regression analysis.

Finally, we composed a flow chart including respondents that completed the IES at the two time points, to gain insight in probable PTSD courses.

## Results

### Study population

During the study period of 30 months, 524 major trauma patients were admitted to the Emergency Department of the study hospital, of which 162 (31%) patients died within 30 days after hospital admission. Of the remaining 362 survivors, 332 were aged 16 years or older. These patients were included in the prospective cohort study on PTSD. One year follow-up measurements of 226 patients (response rate 68%) were obtained. Respondents were significantly older than non-responders (median age 40 versus median age 32, p < 0.05) and the proportion females was higher (non-responders: 18% female versus responders: 34% females, p < 0.05). Trauma mechanism, disturbance of vital parameters, severity of injury or type of pre-hospital trauma care did not differ significantly between responders and non-responders.

**Figure 1:**
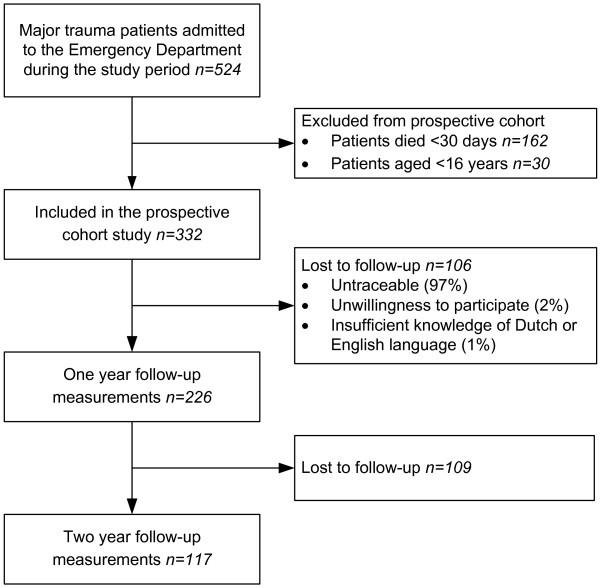
Flow chart of the patient inclusion throughout the study.

Two year follow-up measurements of 117 patients (response rate 52%) were obtained. A flow chart of the patient inclusion throughout the study is shown in Figure 1.

The median age of the respondents was 42 years and 66% were male (Table 1). Female respondents were significantly older than male respondents (median age 49 versus median age 40, p < 0.05).

**Table 1:** Characteristics of the study population patients surviving major trauma at 1 and 2-year follow-up

	**1-year follow-up**		**2-year follow-up**	
	**Returned questionnaire**	**Completed IES**	**Returned questionnaire**	**Completed IES**
N	226	198	117	101
Male^1^	149 (65.9)	132 (65.9)	82 (70.1)	71 (70.3)
Age^2^ (year)	42 (26–59)	41 (24–56)	43 (28–58)	42 (28–57)
Blunt Trauma^1^	218 (96.5)	190 (96.0)	116 (99.1)	100 (99.0)
Glasgow Coma Score^2^	14 (7–15)	14 (8–15)	14 (6–15)	13 (6–15)
Revised Trauma Score^2^	12 (10–12)	12 (10–12)	12 (10–12)	12 (10–12)
Injury Severity Score^2^	22 (17–29)	21 (17–29)	22 (17–30)	22 (17–32)
Prehospital intubation^1^	39 (17.3)	31(15.7)	24 (20.5)	21 (20.8)
Co-morbidity^1^	89 (39.4)	76 (38.4)	42 (35.9)	39 (38.7)

IES = Impact of Event Scale.

^1^patient numbers are displayed, with the percentages given within brackets.

^2^data are displayed as median, with the first and third quartile given within brackets.

The vast majority of patients (97%) sustained a blunt force trauma (non-penetrating trauma). The median ISS of the respondents was 22. The ISS did not differ significantly between female and male respondents (median ISS 20 versus median ISS 22). There were also no significant differences in trauma mechanism, disturbance of vital parameters or type of pre-hospital trauma care between male and female respondents.

### Prevalence of probable PTSD

With reference to the 226 respondents that completed the one year follow-up questionnaire, 198 (88%) filled out the IES. At 2-years follow-up, 101 (86%) respondents filled out the IES. Table 2 shows the characteristics of the respondents an IES-score of 35 or higher, which indicates probable PTSD. At 1-year and 2-year follow-up 23% and 20% respectively of the respondents had probable PTSD.

**Table 2:** **Characteristics, stratified by presence of probable PTSD**^**a**^**(IES-score ≥35)**

**Characteristics**	**1-year follow-up**	**2-year follow-up**
	**Probable PTSD**	**Probable PTSD**
	**46 (23.2%)**	**20 (19.8%)**
*Sociodemographic*		
Gender		
Male	19.7%	15.5%
Female	30.3%	30.0%
Age		
<50	25.5%	19.0%
≥50	17.0%	21.1%
Education		
Primary	27.6%	22.2%
Higher	21.9%	20.0%
Household composition		
Alone	25.9%	21.4%
Not alone	22.6%	20.0%
*Physical*		
Comorbidity		
No comorbidity	14.0%^**^	19.1%
Comorbid disease(s)	37.7%	21.2%
*Injury-related*		
ISS		
<25	22.0%	19.0%
≥25	25.0%	20.9%
Injury localization/AIS^b^		
Head		
<3	31.5%	34.6%^*^
≥3	20.1%	14.7%
Chest		
<3	20.3%	18.5%
≥3	27.5%	21.3%
Abdomen		
<3	21.4%	17.8%
≥3	36.0%	36.4%
Extremities		
<3	19.7%^*^	19.7%
≥3	34.8%	20.0%
Glasgow Coma Scale		
3-8	20.4%	14.3%
9-15	24.3%	22.7%
*Pre-hosp trauma care*		
No EMS	26.3%	23.1%
EMS	21.3%	17.7%

EMS = Emergency Medical Services.

^*^p < 0.05; ^**^p < 0.01.

^a^an IES-score ≥35 signifies symptoms of posttraumatic stress indicative of PTSD.

^b^Injury localization/AIS: patients are dichotomized into two categories: with an AIS below 3 or 3 or higher of a specific injury localization.

### Risk factors for developing PTSD

Univariate logistic regression analyses showed that co-morbid disease and female sex were significantly associated with probable PTSD at one year follow-up. At two years follow-up injuries of the head and extremities were significantly associated with probable PTSD. Multivariate logistic regression analysis including socio-demographic, physical, injury and pre-hospital care variables, indicated that co-morbid disease (OR 4.6; 95% CI, 2.0 to 10.6) and female gender (OR 2.4; 95% CI, 1.1 to 5.2) are strong independent predictors of probable PTSD one year after injury. Head injuries <3 (OR 0.1; 95% CI 0.02 to 0.66) and injuries to the extremities <3 (OR 0.2; 95% CI 0.03 to 0.99) were strong independent predictors of probable PTSD two years after injury.

Pre-hospital trauma care, i.e., assistance of physician staffed Emergency Medical Services (EMS) at the scene of the accident was not significantly associated with probable PTSD at either one or two years follow-up (Table 3).

**Table 3:** **Odds ratios**^a^**(OR) and 95% confidence interval (CI) for the association of probable PTSD (IES-score ≥ 35) with characteristics of the respondent/injury**

	**1-year follow-up**			**2-year follow-up**		
**Characteristics**	**OR**	**95% CI**	**p**	**OR**	**95% CI**	**p**
*Sociodemographic*						
Female sex	2.36	1.06-5.25	<0.05	3.42	0.99-11.75	0.051
Age^b^	2.36	1.06-5.25	0.035	0.98	0.94-1.02	0.242
Primary education	1.38	0.47-3.99	0.558	0.91	0.10-8.36	0.935
HHC: Single	0.88	0.38-2.03	0.756	1.36	0.36-5.17	0.648
*Physical*						
Co-morbidity	4.61	2.02-10.55	<0.01	1.20	0.30-4.89	0.797
*Injury related*						
ISS^b^	0.82	0.28-2.36	0.707	0.46	0.06-3.50	0.449
Injury localization						
Head ≥3	1.02	0.33-3.16	0.969	0.12	0.02-.66	<0.05
Chest ≥3	2.16	0.77-6.09	0.146	1.41	0.22-9.13	0.722
Abdomen ≥3	2.33	0.71-7.66	0.162	2.86	0.44-18.59	0.271
Extremities ≥3	1.68	0.66-4.30	0.276	0.18	0.03-.99	<0.05
Glasgow Coma Scale^b^	0.99	0.90-1.10	0.865	0.94	0.81-1.10	0.444
*Pre-hospital trauma care*^*c*^						
EMS	1.29	0.54-3.06	0.566	0.96	0.27-3.49	0.953

HHC = Household composition, EMS = Emergency Medical Services.

^a^Adjusted for all included predictors in model using multivariate logistic regression analysis.

^b^Continuous variable.

^c^The presence versus absence of pre-hospital trauma care at the scene of the accident via assistance of physician staffed helicopter or other EMS teams.

### Course of PTSD

**Figure 2:**
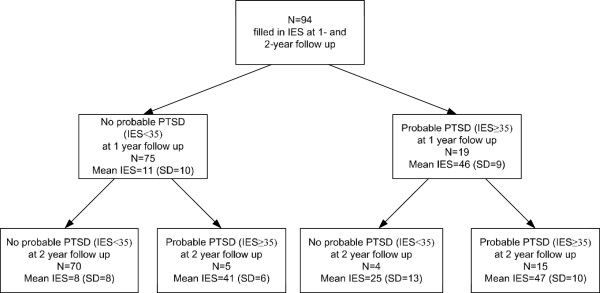
Flowchart of injury patients with and without probable PTSD (IES ≥ 35).

Figure 2 shows the course of probable PTSD over time. This figure includes only those patients who completed the IES at one and two year follow-up (n = 94). Of the patients who did not meet the PTSD criterion of an IES of 35 or higher at 1-year follow-up, 5 (7%) did meet this criterion of PTSD later on. Of these five patients, four had an increase of more than 20 points on the IES scale. One patient had an almost similar IES score at the two time points (32 at 1-year follow-up versus 35 at 2-year follow-up).

Of the patients with symptoms indicative of probable PTSD at one year follow-up, 4 (21%) did not meet the PTSD criterion at two years follow-up. Three of these patients showed a decrease of more than 10 points on the IES scale. Approximately three in four patients (79%; n = 15) had persistent PTSD symptoms indicative of probable PTSD a year later.

## Discussion

PTSD is common in a population of patients with major trauma. One year after trauma 23% of our sample had an IES-score of 35 or higher, indicating probable PTSD. At two years after trauma the prevalence rate of probable PTSD was 20%. Female gender and comorbid disease were strong predictors of probable PTSD one year following injury, whereas head injury and injury of the extremities <3 were strong independent predictors of this disorder at two year follow-up. Of the patients with probable PTSD at one year follow-up 79% had persistent PTSD symptoms a year later.

The prevalence rates that we found in our study are comparable to those found by Kreis et al. [29] and Soberg et al. [11], who reported prevalence rates of 23% and 19% respectively regarding trauma victims with severe injuries (ISS > 15). However, neither Kreis et al. [29] nor Soberg et al. [11] used the IES to assess PTSD, which may have affected the PTSD prevalence rates that were found.

Holbrook et al. [10] did use the IES to assess prevalence of probable PTSD regarding a sample of trauma patients, yet they found a higher prevalence rate of 32% at 18 months follow-up. This difference in prevalence rate might be explained by differences in patient populations.

A second explanation for the differences in prevalence rates of probable PTSD may be the IES cut-off point that was used. Holbrook et al. [10] used an IES-score greater than 24 to identify patients with probable PTSD, whereas in the current study a cut off of 35 was used. When we applied a similar IES cut-off point of 24, the prevalence rate of probable PTSD one year following trauma increased to 36%. At two years follow-up the prevalence rate of probable PTSD increased to 26%. These prevalence rates are in the same range as reported by Holbrook et al. Evidence suggests it is important to use a high IES cut-off score that incurs a high specificity to avoid over diagnosing of PTSD in a comprehensive population with a relative low PTSD prevalence [21,30].

The existence of PTSD symptoms was measured with the IES rather than Clinician-Administered PTSD Scale for DSM-IV (CAPS). Important to note is that the IES is a self-report questionnaire that measures only two of the three main PTSD symptoms, namely intrusion and avoidance. Hyperarousal, the third main PTSD symptom, is not measured by the IES. The IES is not a diagnostic tool, i.e., it is not designed to diagnose mental disorders according to the DSM-IV (the fourth edition of the diagnostic and statistical manual for psychiatric disorders). Consequently, cases that in the current study were identified as having PTSD symptoms might not meet the DSM-IV criteria of clinical PTSD, and inversely. The use of different diagnostic instruments may be a methodological reason for differences in prevalence rates between studies. Presumably, the fact that in this study the IES has been used, and consequently PTSD symptoms excluding hyperarousal symptoms have been assessed may have resulted in a relatively high rate of probable PTSD.

In this study, a quarter of the sample met criteria for a moderate TBI, and another 20% met criteria for a severe TBI. TBI can have serious effects on communication and cognition, and the presence of severe TBI will impact upon any assessment of PTSD. Moreover, a large prospective cohort investigation of injured trauma survivors with TBI in the United States found an elevated risk of PTSD among patients with mild TBI as more than one in five (22%) was diagnosed with PTSD at one year follow up [31].

Our study focused on a single stressor, i.e., injury, whereas PTSD generally originates from cumulative exposure to traumatic stressors. The presence of traumatic stressors also influences the probability of spontaneous remission from PTSD [32,33]. This means that the level of other traumatic stressors may affect to a large extent the prevalence rates that were found. At long-term follow-up (>1 year) PTSD prevalence rates from 5% [9] to 32% [10] have been reported regarding trauma patients. This variety in PTSD prevalence rates can therefore be explained by differences in exposure to traumatic stressors other than injury.

To identify subgroups at risk for long-term probable PTSD, risk factors for the development of probable PTSD one and two year following injury were assessed. The results of our study indicate a strong association between female gender and probable PTSD. This association is in line with findings in the general literature [34] and has been reported by other studies on PTSD following injuries [10,35-37]. In literature several explanations were found for these gender differences in PTSD risk, such as women’s stronger perceptions of threat and loss of control and higher levels of peritraumatic dissociation, as well as gender-specific acute psychobiological reactions to trauma [34]. Another independent predictor of probable PTSD at one year follow-up was the presence of one or more co-morbid diseases. Severity of the injury, reflected by the ISS, was not significantly associated with probable PTSD. This is in accordance with findings from previous studies [11,12,29,38]. However, a limitation of our study is that the comorbidity measure is very simple, since it groups comorbidities into none, one or more than one. No distinction was made whether the comorbidities are related to pre-existing psychiatric disorders and/or substance abuse. If this distinction was made, it would shed more light on the relation between different types of co-morbidity and PTSD.

Furthermore, peritraumatic processing, social support, peritraumatic dissociation or other predictors of PTSD were not included in this study [39,40].

Pre-hospital trauma care, i.e., the presence of helicopter or other physician staffed EMS teams at the scene of the accident was not significantly associated with probable PTSD at either one or two years follow-up. Dispatch is based on the apparent seriousness of a distress call or trauma mechanism, or based on patients’ condition as assessed by ambulance personnel at the scene of the accident. In other words, helicopter or other physician staffed EMS teams attend to the most severe cases and specific trauma mechanism. Our hypothesis was that cases with pre-hospital trauma care at the scene may be more prone to develop PTSD. The finding that pre-hospital care was not significantly associated with PTSD may be explained by the finding that trauma mechanism, disturbance of vital parameters or severity of injury are not significantly associated with probable PTSD.

The results of this study may not be generalizable to young male major trauma patients, because respondents who were willing to participate in the study were significantly older and significantly more likely to be female.

A strength of this study was that it did not focus solely on prevalence rates of probable PTSD, but also addressed the course of probable PTSD at long-term follow-up. Previous research on the course of PTSD revealed that patients experience symptoms for one year or longer [41,42]. The flowchart depicting injury patients with and without symptoms at one and two years following injury allowed us to gain insight into the development of probable PTSD over time in a sample of severely injured trauma patients. The flowchart showed that PTSD symptoms fluctuate over time; patients meet criteria or cut-off at some time points, and at other points they fell just below, but that the majority of patients with probable PTSD at one year follow-up still meet the IES criterion for PTSD at two years follow-up. This indicates that many patients might suffer from prolonged effects of PTSD.

Most likely PTSD symptoms occur during the first year after the accident, rather than between year one and two after the injury. However, in the current study baseline information on PTSD symptoms was not gathered. As a result, the course of probable PTSD in the first year after the injury could not be analyzed, nor does this study provide information about pre-existing PTSD.

## Conclusions

We conclude that almost one in four major trauma patients have an IES-score of 35 or higher, indicating evident symptoms of PTSD one year after sustaining the injuries. At two years follow-up almost one in five major trauma patients suffered from probable PTSD. Female gender and co-morbidity were the strongest independent predictors of this disorder. Research on the course of PTSD symptoms showed that major trauma patients can develop and recover from probable PTSD at different time points, but the majority of patients with probable PTSD at one year follow-up still met the IES criterion for probable PTSD at two years follow-up. Since PTSD raises a major and often prolonged barrier for full recovery of injury patients, the development and evaluation of ED and hospital-based policies for early diagnosis and treatment of PTSD should be stimulated.

## Abbreviations

AIS: Abbreviated injury scale; CAPS: Clinician-administered PTSD scale for DSM-IV; CI: Confidence interval; ED: Emergency department; EMS: Emergency medical services; IES: Impact of event scale; ISS: Injury severity score; OR: Odds ratio; PTSD: Posttraumatic stress disorder.

## Competing interests

We declare that there are no financial or non-financial competing interests (political, personal, religious, ideological, academic, intellectual, commercial or any other) to declare in relation to this manuscript.

## Authors’ contributions

JAH executed the statistical analysis and drafted the manuscript. ANR participated in the design of the study, data collection and drafting of the manuscript. EMMvL assisted with the statistical analysis and drafting of the manuscript. EFvB participated in the design of study and drafting of the manuscript. PP participated in the design of study and drafting of the manuscript. IBS participated in the design of study and drafting of the manuscript. SP supervised, participated in the design of study, assisted with the statistical analysis and drafting of the manuscript. All authors read and approved the final manuscript.

## Pre-publication history

The pre-publication history for this paper can be accessed here:

http://www.biomedcentral.com/1471-244X/12/236/prepub

## References

[B1] Polinder S Haagsma JA Toet H Brugmans MJ van Beeck EF Burden of injury in childhood and adolescence in 8 European countries BMC Publ Health 2010 10 45 10.1186/1471-2458-10-45 PMC2824737 20113463

[B2] Holtslag HR Post MW Lindeman E Van der Werken C Long-term functional health status of severely injured patients Injury 2007 38 3 280 289 10.1016/j.injury.2006.10.026 17250834

[B3] Ringburg AN Polinder S van Ierland MC Steyerberg EW van Lieshout EM Patka P van Beeck EF Schipper IB Prevalence and prognostic factors of disability after major trauma J Trauma 2011 70 4 916 922 10.1097/TA.0b013e3181f6bce8 21045741

[B4] Vles WJ Steyerberg EW Essink-Bot ML van Beeck EF Meeuwis JD Leenen LP Prevalence and determinants of disabilities and return to work after major trauma J Trauma 2005 58 1 126 135 10.1097/01.TA.0000112342.40296.1F 15674163

[B5] Davidson JR Stein DJ Shalev AY Yehuda R Posttraumatic stress disorder: acquisition, recognition, course, and treatment J Neuropsychiatry Clin Neurosci 2004 16 2 135 147 10.1176/appi.neuropsych.16.2.135 15260364

[B6] de Vries GJ Olff M The lifetime prevalence of traumatic events and posttraumatic stress disorder in the Netherlands J Trauma Stress 2009 22 4 259 267 10.1002/jts.20429 19645050

[B7] Kessler RC Posttraumatic stress disorder: the burden to the individual and to society J Clin Psychiatry 2000 61 Suppl 5 4 12 discussion 13–14 10761674

[B8] Blanchard EB Hickling EJ Taylor AE Loos WR Forneris CA Jaccard J Who develops PTSD from motor vehicle accidents? Behav Res Ther 1996 34 1 1 10 10.1016/0005-7967(95)00058-6 8561759

[B9] Mayou R Tyndel S Bryant B Long-term outcome of motor vehicle accident injury Psychosom Med 1997 59 6 578 584 9407575 10.1097/00006842-199711000-00004

[B10] Holbrook TL Hoyt DB Stein MB Sieber WJ Gender differences in long-term posttraumatic stress disorder outcomes after major trauma: women are at higher risk of adverse outcomes than men J Trauma 2002 53 5 882 888 10.1097/00005373-200211000-00012 12435938

[B11] Soberg HL Bautz-Holter E Roise O Finset A Mental health and posttraumatic stress symptoms 2 years after severe multiple trauma: self-reported disability and psychosocial functioning Arch Phys Med Rehabil 2010 91 3 481 488 10.1016/j.apmr.2009.11.007 20298843

[B12] Harris IA Young JM Rae H Jalaludin BB Solomon MJ Predictors of post-traumatic stress disorder following major trauma ANZ J Surg 2008 78 7 583 587 10.1111/j.1445-2197.2008.04578.x 18593415

[B13] Holbrook TL Hoyt DB Stein MB Sieber WJ Perceived threat to life predicts posttraumatic stress disorder after major trauma: risk factors and functional outcome J Trauma 2001 51 2 287 292 discussion 292–283 10.1097/00005373-200108000-00010 11493786

[B14] Bisson JI Shepherd JP Joy D Probert R Newcombe RG Early cognitive-behavioural therapy for post-traumatic stress symptoms after physical injury. Randomised controlled trial Br J Psychiatry 2004 184 63 69 10.1192/bjp.184.1.63 14702229

[B15] Baker SP O’Neill B Haddon W Jr Long WB The injury severity score: a method for describing patients with multiple injuries and evaluating emergency care J Trauma 1974 14 3 187 196 10.1097/00005373-197403000-00001 4814394

[B16] Champion HR Copes WS Sacco WJ Lawnick MM Keast SL Bain LW Jr Flanagan ME Frey CF The major trauma outcome study: establishing national norms for trauma care J Trauma 1990 30 11 1356 1365 10.1097/00005373-199011000-00008 2231804

[B17] Teasdale G Jennett B Assessment of coma and impaired consciousness. A practical scale Lancet 1974 2 7872 81 84 4136544 10.1016/s0140-6736(74)91639-0

[B18] Champion HR Sacco WJ Copes WS Gann DS Gennarelli TA Flanagan ME A revision of the trauma score J Trauma 1989 29 5 623 629 10.1097/00005373-198905000-00017 2657085

[B19] Ringburg AN Polinder S Meulman TJ Steyerberg EW van Lieshout EM Patka P van Beeck EF Schipper IB Cost-effectiveness and quality-of-life analysis of physician-staffed helicopter emergency medical services Br J Surg 2009 96 11 1365 1370 10.1002/bjs.6720 19847879

[B20] Horowitz M Wilner N Alvarez W Impact of event scale: a measure of subjective stress Psychosom Med 1979 41 3 209 218 472086 10.1097/00006842-197905000-00004

[B21] Wohlfarth TD van den Brink W Winkel FW ter Smitten M Screening for posttraumatic stress disorder: an evaluation of two self-report scales among crime victims Psychol Assess 2003 15 1 101 109 12674729 10.1037/1040-3590.15.1.101

[B22] van der Ploeg E Mooren TT Kleber RJ van der Velden PG Brom D Construct validation of the Dutch version of the impact of event scale Psychol Assess 2004 16 1 16 26 15023089 10.1037/1040-3590.16.1.16

[B23] Holbrook TL Anderson JP Sieber WJ Browner D Hoyt DB Outcome after major trauma: 12-month and 18-month follow-up results from the trauma recovery project J Trauma 1999 46 5 765 771 10.1097/00005373-199905000-00003 10338392

[B24] Holbrook TL Hoyt DB The impact of major trauma: quality-of-life outcomes are worse in women than in men, independent of mechanism and injury severity J Trauma 2004 56 2 284 290 10.1097/01.TA.0000109758.75406.F8 14960969

[B25] Polinder S van Beeck EF Essink-Bot ML Toet H Looman CW Mulder S Meerding WJ Functional outcome at 2.5, 5, 9, and 24 months after injury in the Netherlands J Trauma 2007 62 1 133 141 10.1097/TA.0b013e31802b71c9 17215744

[B26] Mosby Mosby’s Medical dictionary 2009 8th Elsevier Health Sciences

[B27] The abbreviated injury scale 1990 revision, update 98 1998 Des Plaines, IL: Association for the Advancement of Automotive Medicine

[B28] Rubin DB Schenker N Multiple imputation in health-care databases: an overview and some applications Stat Med 1991 10 4 585 598 10.1002/sim.4780100410 2057657

[B29] Kreis BE Castano NJY Tuinebreijer WE Hoogenboom LCA Meylaerts SAG Rhemrev SJ Characteristics of polytrauma patients with posttraumatic stress disorder in a level 1 trauma center Eur J Trauma Emerg Surg 2011 37 3 269 275 10.1007/s00068-011-0109-2 26815109

[B30] Neal LA Busuttil W Rollins J Herepath R Strike P Turnbull G Convergent validity of measures of post-traumatic stress disorder in a mixed military and civilian population J Trauma Stress 1994 7 3 447 455 10.1002/jts.2490070310 8087405

[B31] Zatzick DF Rivara FP Jurkovich GJ Hoge CW Wang J Fan MY Russo J Trusz SG Nathens A Mackenzie EJ Multisite investigation of traumatic brain injuries, posttraumatic stress disorder, and self-reported health and cognitive impairments Arch Gen Psychiatry 2010 67 12 1291 1300 10.1001/archgenpsychiatry.2010.158 21135329 PMC3102494

[B32] Lloyd DA Turner RJ Cumulative adversity and posttraumatic stress disorder: evidence from a diverse community sample of young adults Am J Orthopsychiatry 2003 73 4 381 391 14609400 10.1037/0002-9432.73.4.381

[B33] Turner RJ Lloyd DA Lifetime traumas and mental health: the significance of cumulative adversity J Health Soc Behav 1995 36 4 360 376 10.2307/2137325 8719054

[B34] Olff M Langeland W Draijer N Gersons BP Gender differences in posttraumatic stress disorder Psychol Bull 2007 133 2 183 204 17338596 10.1037/0033-2909.133.2.183

[B35] Breslau N Davis GC Andreski P Peterson EL Schultz LR Sex differences in posttraumatic stress disorder Arch Gen Psychiatry 1997 54 11 1044 1048 10.1001/archpsyc.1997.01830230082012 9366662

[B36] Mayou R Bryant B Consequences of road traffic accidents for different types of road user Injury 2003 34 3 197 202 10.1016/S0020-1383(02)00285-1 12623250

[B37] Fullerton CS Ursano RJ Epstein RS Crowley B Vance K Kao TC Dougall A Baum A Gender differences in posttraumatic stress disorder after motor vehicle accidents Am J Psychiatry 2001 158 9 1486 1491 10.1176/appi.ajp.158.9.1486 11532736

[B38] Quale AJ Schanke AK Froslie KF Roise O Severity of injury does not have any impact on posttraumatic stress symptoms in severely injured patients Injury 2009 40 5 498 505 10.1016/j.injury.2008.11.006 19332345

[B39] Johansen VA Wahl AK Eilertsen DE Weisaeth L Prevalence and predictors of post-traumatic stress disorder (PTSD) in physically injured victims of non-domestic violence. A longitudinal study Soc Psychiatry Psychiatr Epidemiol 2007 42 7 583 593 10.1007/s00127-007-0205-0 17530151

[B40] Ozer EJ Best SR Lipsey TL Weiss DS Predictors of posttraumatic stress disorder and symptoms in adults: a meta-analysis Psychol Bull 2003 129 1 52 73 12555794 10.1037/0033-2909.129.1.52

[B41] Davidson JR Hughes D Blazer DG George LK Post-traumatic stress disorder in the community: an epidemiological study Psychol Med 1991 21 3 713 721 10.1017/S0033291700022352 1946860

[B42] Breslau N Outcomes of posttraumatic stress disorder J Clin Psychiatry 2001 62 Suppl 17 55 59 11495098

